# Consequences of Both Coxsackievirus B4 and Type 1 Diabetes on Female Non-Obese Diabetic Mouse Kidneys

**DOI:** 10.3390/microorganisms9112357

**Published:** 2021-11-15

**Authors:** Debra L. Walter, Jean R. Thuma, Ramiro Malgor, Frank L. Schwartz, Kelly D. McCall, Karen T. Coschigano

**Affiliations:** 1 Interdisciplinary Program in Molecular and Cellular Biology, Ohio University, Athens, OH 45701, USA; malgor@ohio.edu (R.M.); mccallk@ohio.edu (K.D.M.); coschigk@ohio.edu (K.T.C.); 2 Department of Biological Sciences, College of Arts and Sciences, Ohio University, Athens, OH 45701, USA; 3 Department of Specialty Medicine, Heritage College of Osteopathic Medicine, Ohio University, Athens, OH 45701, USA; watts@ohio.edu (J.R.T.); endoguy.ou@gmail.com (F.L.S.); 4 Department of Biomedical Sciences, Heritage College of Osteopathic Medicine, Ohio University, Athens, OH 45701, USA; 5 The Diabetes Institute, Heritage College of Osteopathic Medicine, Ohio University, Athens, OH 45701, USA; 6 Translational Biomedical Sciences Program, Heritage College of Osteopathic Medicine, Ohio University, Athens, OH 45701, USA; 7 Biomedical Engineering Program, Russ College of Engineering and Technology, Ohio University, Athens, OH 45701, USA

**Keywords:** kidney injury profiling, coxsackievirus and type 1 diabetes, risk factors for kidney disease

## Abstract

Despite the 2019 Executive Order on Advancing American Kidney Health Initiative, kidney disease has moved up in rank from the 9th to the 8th leading cause of death in the United States. A recent push in the field of nephrology has been to identify molecular markers and/or molecular profiles involved in kidney disease process or injury that can help identify the cause of injury and predict patient outcomes. While these studies have had moderate success, they have not yet considered that many of the health conditions that cause kidney disease (diabetes, hypertension, etc.) can also be caused by environmental factors (such as viruses), which in and of themselves can cause kidney disease. Thus, the goal of this study was to identify molecular and phenotypic profiles that can differentiate kidney injury caused by diabetes (a health condition resulting in kidney disease) and coxsackievirus B4 (CVB4) exposure (which can cause diabetes and/or kidney disease), both alone and together. Non-obese diabetic (NOD) mice were used for this study due to their susceptibility to both type 1 diabetes (T1D)- and CVB4-mediated kidney injury, in order to glean a better understanding of how hyperglycemia and viral exposure, when occurring on their own and in combination, may alter the kidneys’ molecular and phenotypic profiles. While no changes in kidney function were observed, molecular biomarkers of kidney injury were significantly up- and downregulated based on T1D and CVB4 exposure, both alone and together, but not in a predictable pattern. By combining individual biomarkers with function and phenotypic measurements (i.e., urinary albumin creatinine ratio, serum creatinine, kidney weight, and body weight), we were able to perform an unbiased separation of injury group based on the type of injury. This study provides evidence that unique kidney injury profiles within a kidney disease health condition are identifiable, and will help us to identify the causes of kidney injury in the future.

## 1. Introduction

Kidney disease is the 8th leading cause of death in the United States, costing Medicare in excess of USD 120 billion a year on chronic kidney disease (CKD) and end-stage renal disease (ESRD) alone [1]. While individual healthcare costs for patients are exorbitant, the impact on a patient’s quality of life is poor. Making matters worse, it is estimated that 9 out of 10 patients with CKD do not know they have kidney disease [2], while one-third of patients diagnosed with ESRD—kidney failure requiring dialysis—had no prior nephrology care prior to kidney failure [3]. The question arises: when there are five progressive stages to chronic kidney disease, why are so many patients unaware of the severity of their situation until their kidneys fail? While patient lifestyle and awareness play a large role in disease outcomes [4,5,6,7], the answer may be more complicated, and rooted in our current understanding of individual disease progression. Risk factors for developing CKD include diabetes, hypertension, family history, smoking, obesity, age, and race/ethnicity [8,9]; however, just because an individual is high risk does not mean that they will develop kidney disease, and likewise, individuals without any of these risk factors can still develop kidney disease. In addition to simply being risk factors for developing CKD, many health conditions can cause kidney damage and lead to CKD. While diabetes and hypertension are the two main health conditions that cause CKD, numerous other conditions—such as glomerulonephritis, polycystic kidney disease, cancer, lupus, renal and urinary tract obstructions, repeated urinary tract infections, and a variety of rare diseases—can all cause long-term kidney damage and subsequent CKD [10]. Making matters more complicated is the fact that viruses have been implicated as possible causative agents for many of the risk factors and health conditions for developing CKD, including diabetes, hypertension, cystic kidney, and cancer [11,12,13,14,15,16]. Consideration of viral infections, which can be ubiquitous and seemingly innocuous, may provide insight into why some patients develop kidney injury and subsequent disease, while others do not.

The kidneys function to secrete hormones and filter waste from the blood, while retaining essential ions and small molecules. CKD develops as a slow-progressing, permanent kidney injury, eventually leading to renal failure. Kidney injury includes arterial hardening; basement membrane thickening; mesangial cell proliferation, expansion, and sclerosis; collagen deposition; podocyte effacement or loss; tubular epithelial cell atrophy; and/or immune cell infiltration [17,18]. The most widely accepted clinical measurement to classify the stage of kidney disease and, thus, kidney function, is the glomerular filtration rate (GFR), which ranges from ≥90 mL/min (stage 1; mild kidney damage with normal kidney function) to ≤15 mL/min (stage 5; kidney failure) [19]. While assessing GFR is preferred, it is difficult to measure in clinical practice due to the requirement of a 24 h urine collection and, thus, is not always practical. Other measures of kidney function that can be measured accurately in the clinic include urinary albumin to creatinine ratio (UACR), serum creatinine (SrCr), and blood urea nitrogen (BUN), all of which have standard ranges that indirectly measure kidney function [20,21]. UACR typically increases with kidney injury, indicating that albumin either is leaking through the glomerular filtration barrier into the urine (glomerular injury), or is not being efficiently reabsorbed by the tubules (tubular injury). Creatinine and urea are normal waste products from muscle metabolism and protein breakdown, respectively, that are excreted by the kidneys. When kidney function declines, both SrCr and BUN will become elevated. 

While GFR, UACR, SrCr, and BUN are common clinical measures to determine kidney function, they only provide insight into how severe the kidney damage is, and not the cause of the damage. There has been a recent push in the field of nephrology to identify molecular markers to help identify the cause of injury, determine kidney disease progression, and predict patient outcomes. The field has had moderate success in identifying molecular markers indicative of glomerular and tubular health, inflammation, and fibrosis [22,23]; however, many of these biomarkers are involved in multiple types of injuries and, thus, still only identify that there is a problem, and not what that problem is. For example, KIM1 is upregulated in proximal tubule cells following injury; however, injuries that result in KIM1 upregulation include drug-induced toxicity, diabetic nephropathy, hypoxia, and fibrosis [24,25,26,27,28,29,30]; thus, it has been difficult to determine the cause of injury from a single molecular marker. Large-scale molecular profiling may be able to help combat this problem by identifying combinatorial molecular markers (molecular profiles) involved in one disease process or injury that are different from another disease process. Together, individual disease- or injury-related molecular and phenotypic profiles may be more powerful in identifying subtle changes in kidney health and unique causes of kidney injury, and may help us to understand why some patients are more susceptible to kidney disease than others, even when both patients are in the same high-risk category. 

In order to identify how two insults that are involved in kidney injury may result in insult-specific profiles, both individually and combined, we considered diabetes—the number one health condition to cause kidney disease—and coxsackievirus B4 (CVB4), an enterovirus that can initiate the onset of type 1 diabetes (T1D), and can also result in kidney disease in the absence of hyperglycemia [31,32,33,34]. Prolonged exposure of the kidneys to glucose, as occurs with T1D, results in kidney function decline due to gradual overload of the electron transport chain, leading to the production of reactive oxygen species, growth factors and cytokines, advanced glycation end-products, extracellular matrix accumulation, and glomerulosclerosis and tubulointerstitial fibrosis [35]. CVB4 can initiate acute beta-cell destruction and diabetes that lacks an autoimmune component in genetically susceptible individuals, likely via the bystander effect or molecular mimicry [13,36,37]. However, injury in the kidney resulting from CVB4 exposure is much less defined than in the pancreas, due to the seemingly innocuous nature of the virus. CVB4 can infect at least three cell types of the kidney—podocytes, mesangial cells, and tubular epithelial cells [38]—and has been responsible for a number of kidney-failure-related deaths [31,39]. However, the current state of our knowledge relies on studies using human cell cultures or serial infections of CVB4 [40]. We have previously demonstrated that a one-time exposure to CVB4 in NOD mice in the absence of hyperglycemia resulted in elevated expression of pattern recognition receptor response genes, some of which remained elevated at least 14 days post-exposure [33]. Moreover, we demonstrated that a one-time CVB4 exposure resulted in increased glomerular scarring 17 weeks post-exposure when compared to non-exposed mice, suggesting that early gene expression changes may result in long-term injury that was previously undefined. While both T1D and CVB4 are known instigators of kidney injury in humans and mice, little work has been done to characterize the molecular and phenotypic profiles of each insult (hyperglycemia and CVB4 exposure) when occurring separately and together, or to relate the profiles to subsequent kidney injury. Thus, the purpose of this study was to describe the kidney injury occurring from hyperglycemia and CVB4 exposure, both alone and together, along with identifying insult-specific profiles based on kidney gene expression and physiological changes that may be unique to each insult, in order to better understand how each contributes to kidney injury. 

The non-obese diabetic (NOD) mouse model is widely used to study T1D, due to its genetic predisposition to spontaneously develop T1D. Furthermore, it can develop albuminuria, mesangial proliferation, and sclerosis following hyperglycemia, serving as a model of diabetic kidney injury [41]. Moreover, the NOD mouse is susceptible to CVB4-mediated kidney injury, including a pattern recognition response, increased mesangial area, and gene expression changes [33]. By combining the NOD mouse model and CVB4 exposure, we can study the contribution of both hyperglycemia and CVB4 to kidney injury. We hypothesized that CVB4 and T1D, both alone and together, would result in similar but distinguishable molecular and phenotypic kidney profiles (gene expression, morphometric, and kidney function parameters). This knowledge should be useful to more accurately identify patients at risk of developing kidney injury, as well as the causes of kidney injury.

## 2. Materials and Methods

### 2.1. Animal Procedure Approvals

The animal experiment was performed in accordance with the Association for Assessment and Accreditation of Laboratory Animal Care (AAALAC) guidelines and standards set by federal, state, and local authorities, and approved by the Ohio University Institutional Animal Care and Use Committee (IACUC) under protocol number 13-H-043. Ohio University’s animal care program is maintained in accordance with Public Health Service (PHS) policy, and meets the standards for care and housing set by the “Guide for the Care and Use of Animals” published by the National Research Council. The program also maintains accordance with all regulations of the United States Department of Agriculture. The animal care program is fully accredited by the Association for the Assessment and Accreditation of Laboratory Animal Care, International (AAALAC, Int.).

### 2.2. Animal Procedures

Eight-week-old female NOD/ShiLtJ (NOD) mice were purchased from the Jackson Laboratories (001976, The Jackson Laboratories, Bar Harbor, ME, USA) and used for this study, due to their higher incidence of T1D development as they age (50% of females compared to 30% of males by 22 weeks of age) [42,43,44]. Coxsackievirus B4 (CVB4) Edwards strain (GenBank: S76772.1) was used for this study (a generous gift from Roger Loria, Virginia Commonwealth University, Richmond, VA, USA). NOD mice were exposed at 10 weeks of age via intraperitoneal (IP) injection of 500,000 plaque-forming units (PFUs) of CVB4 or sterile phosphate-buffered saline (PBS) (#J373, VWR, Radnor, PA, USA) as a stress control. Body weight and blood glucose were measured once a week for the course of the study. A drop of blood was collected from the tip of the tail and applied to a handheld glucometer (Freestyle Freedom Lite Blood Glucose Monitoring System, Abbott Laboratories, Lake Bluff, IL, USA). Mice were considered to be diabetic with a non-fasting blood glucose level ≥ 250 mg/dL, and confirmed by glucosuria prior to euthanasia (#2806, Bayer Healthcare, Leverkusen, Germany).

### 2.3. Experimental Design

**Figure 1:**
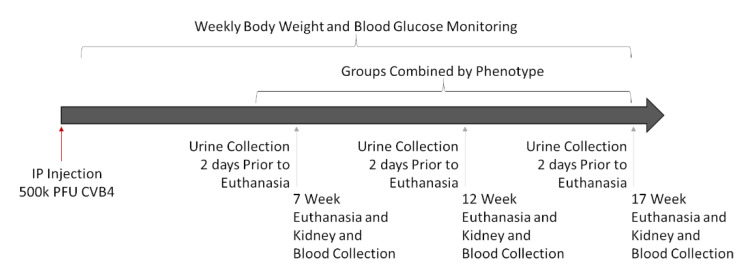
Graphical workflow of animal study: Eighteen female NOD mice were IP-injected with 500,000 PFUs of CVB4 at 10 weeks of age. Mice were euthanized at 7, 12, and 17 weeks post-injection, and assessed as described below. Three timepoints were combined into one group per phenotype (V(−)ND, V(−)DB, V(+)ND, and V(+)DB), because blood glucose and CVB4 were not dependent on time.

Experiments were designed to assess kidney damage at progressive timepoints (7, 12, and 17 weeks post-infection) to allow for the comparison of signs of kidney damage among CVB4-exposed and -non-exposed mice with and without diabetes. Multiple timepoints were included for two reasons: (1) diabetes develops in NOD mice over time (and at different times in different mice), and (2) signs of diabetic (and potentially viral) kidney damage can take time to develop—some indications, such as increased kidney weight and *Tgfb* and *Cd68* gene expression, begin a few days after diabetes is first detected [45,46,47], but others, such as elevated SrCr, BUN, mesangial expansion, collagen deposition, and fibrosis, take more time [19,45,48]. Comparison of the four groups of mice—non-exposed/non-diabetic (V(−)ND), non-exposed/diabetic (V(−)DB), CVB4-exposed/non-diabetic (V(+)ND) and CVB4-exposed/diabetic (V(+)DB)—provided the information necessary to identify kidney damage due to each causative agent (diabetes and virus exposure), both alone and together. A graphical workflow can be seen in Figure 1.

### 2.4. Urine Collection

Two days prior to euthanasia, two-hour urine collection was performed, as previously described [49,50]. Briefly, each mouse was placed in a clean individual cage (#M-BTM, Innovive, Hudson, NH, USA) with no food, water, or bedding for two hours. At 15-min intervals, urine was collected from the cage bottom with a pipette. Urine was stored on ice until centrifugation at 1000× *g* for 5 min, and then the supernatant was transferred to a new tube and stored at −80 °C. 

### 2.5. Blood Collection

Blood was collected via cardiac puncture under anesthesia at the time of euthanasia. Once properly anesthetized, approximately 1 mL of blood was collected via cardiac puncture (#329652, BD Biosciences, San Jose, CA, USA) [51] from each animal, and allowed to clot at room temperature for 10 min. Samples were then stored on ice until centrifugation at 5000× *g* for 10 min, and serum was transferred to a new tube and stored at −80 °C.

### 2.6. Kidney Collection

After euthanasia, both kidneys were collected, the capsule removed by gentle rubbing with a KimWipe (#06-666, Kimberly-Clark, West Chester, OH, USA), and the left kidney weight recorded. Left kidney poles were removed, flash-frozen with the whole right kidney in liquid nitrogen, and stored at −80 °C. The remainder of the left kidney was prepared for histology as described below.

### 2.7. Kidney Histology

At euthanasia, kidney tissue was fixed in 10% neutral buffered formalin (#SF98-20, Fisher Scientific, Hampton, NH, USA) for 24 h and embedded in paraffin (#EM-400, Leica Biosystems Inc., Buffalo Grove, IL, USA). Then, 12- and 17-week formalin-fixed, paraffin-embedded (FFPE) kidney sections (4 μm) were mounted on SuperFrost Plus glass microscope slides (22-037-246, Thermo Fisher, Waltham, MA, USA) and stained at the Ohio University histology core facility with periodic acid–Schiff (PAS) without diastase. Tissue sections were also stained with Masson’s trichrome (trichrome) (#HT15, Sigma-Aldrich, St. Louis, MO, USA), following the manufacturer’s standard procedure, with the following alterations: Following rehydration, samples were mordanted in Bouin’s solution (#HT10132, Sigma-Aldrich, St. Louis, MO, USA) for 5 h at room temperature. Slides were dehydrated and cleared by dipping in and out of each reagent for 15 s, using a regular cadence, and mounted with Permount (#SP15, Thermo Fisher, Waltham, MA, USA). To evaluate mesangial matrix expansion, images of five glomeruli per PAS sample were taken at 400× magnification. Individual glomeruli were isolated from images, and total PAS-positive (pink) area was measured using ImageJ v.1.48 color threshold analysis, divided by total glomerular area measured with the freehand selection tool, and expressed as a percentage [52]. To evaluate immune cell infiltration, three 4 μm sections obtained 150 μm apart were stained with PAS as described above. Images were taken at 200× magnification of all areas with visible immune cell infiltration. The ImageJ freehand selection tool was used to select and measure each immune cell infiltration area on each image. Raw infiltration area numbers were first analyzed according to group, without manipulation. Total infiltration area for one mouse was then calculated by adding the infiltration areas from all three sections together. Total number of infiltration regions was also calculated by counting each region of infiltration per animal (total number of regions per three images). If identification of an immune cell infiltration area was questionable, H&E staining was used for confirmation, but not for analysis. Trichrome staining was used to detect collagen deposition (blue staining), and noted as presence/absence. Representative images of trichrome staining were taken at 400× magnification. All images were taken on a Nikon Eclipse 80i microscope (Nikon, Tokyo, Japan) using Image-Pro Software (Media Cybernetics, Rockville, MD, USA), and analysis was performed using ImageJ v.1.48 software [52]. 

### 2.8. Gene Expression Analysis

RNA was isolated from a portion (~20 mg) of flash-frozen whole kidney using RNA STAT-60 (#CS-110, Tel-Test, Inc., Friendswood, TX, USA) and a Bullet Blender homogenizer with RNase-free 0.5 mm ZrO_2_ beads (Next Advance, Inc., Troy, NY, USA). DNase treatment and RNA cleanup were performed using an RNA Clean and Concentrator kit with included DNase, following the manufacturer’s protocol (#R1016, Zymo Research, Tustin, CA, USA), using 15 μg of RNA. Reverse transcription of 2 μg of RNA was performed using a High-Capacity cDNA Reverse Transcription kit (#4368814, Life Technologies, Carlsbad, CA, USA). RT-qPCR was performed using iTaq Universal SYBR Green SuperMix (#172-5122, Bio-Rad Laboratories, Inc., Hercules, CA, USA) or TaqMan Universal Gene Expression Master Mix (#4370074, Applied Biosystems, Foster City, CA, USA) and a StepOne Plus Real-Time PCR System (Applied Biosystems, Waltham, MA, USA) or CFX384 (Bio-Rad, Hercules, CA, USA). Gene selection was based on a literature search of the most common kidney injury biomarkers known to be up- or downregulated during kidney injury [53,54,55,56]. A literature search on each biomarker was performed to determine its utility as a biomarker of hyperglycemia or viral-mediated kidney injury. Eighteen well-described RNA kidney injury biomarkers of hyperglycemia, viral exposure, proinflammatory immune response, or general kidney disease biomarkers comprised the final list. All SYBR Green and TaqMan primers are listed in Supplementary Tables S1 and S2, respectively. All SYBR green assays were normalized to the geometric mean of glyceraldehyde 3-phsophate dehydrogenase (*Gapdh*) and actin gamma 1 (*Actg1*), and all TaqMan assays were normalized to *Gapdh* based on previous control gene analysis [57]. 

### 2.9. Urine Analysis

Urine albumin and creatinine were measured according to the manufacturers’ protocols using a murine albumin ELISA (#E90-134, Bethyl Laboratories, Montgomery, TX, USA) and the creatinine companion (#1012, Exocell, Philadelphia, PA, USA), respectively; both assays use a standard curve to calculate concentration (μg/mL for albumin and mg/mL for creatinine). The μg/mg urinary albumin/creatinine ratio (UACR) was then calculated by dividing albumin by creatinine.

### 2.10. Serum Analysis

Murine sera collected at the time of euthanasia were sent to the University of Massachusetts Mouse Metabolic Phenotyping Core for evaluation of serum creatinine (SrCr) concentration (Worchester, MA, USA) using a Cobas Clinical Chemistry Analyzer (Roche, Basel, Switzerland). 

### 2.11. Statistical Analyses

All statistical analyses were performed using R Statistical software [58] (Vienna, Austria) and graphed using GraphPad Prism (v.9.2.0, San Diego, CA, USA). The Shapiro–Wilk test and Levene’s test were used to test for normality and equal variance, respectively. Data not falling within a normal distribution were transformed to best approach normality. Student’s *t*-test was used to compare the means of V(+)ND and V(+)DB CVB4 abundance. Main effects were tested using two-way ANOVA, with CVB4 exposure and diabetes as factors. Tukey’s post-hoc test was performed when a main effect was found. To test for significant correlations, Pearson’s correlation coefficient was calculated. Non-metric multidimensional scaling (NMDS) of Bray–Curtis dissimilarity (stress ~0.1) was used as an exploratory approach for group separation of non-parametric data, followed by a permutational multivariate analysis of variance (PERMANOVA). For all statistical tests, alpha was set at 0.05.

## 3. Results

### 3.1. NOD Mouse Group Characteristics at Time of Euthanasia

To carry out the current study, female NOD mice were exposed to CVB4 at 10 weeks of age. Supplementary Table S3 indicates the numbers of NOD mice in each group at the time of euthanasia after virus exposure, as well as the time (in weeks) that mice were diabetic (where applicable). Initial data exploration and variation in sample size made it difficult to draw definitive conclusions for any of the groups at any individual timepoint; therefore, animals from the three timepoints—7, 12, and 17 weeks post-exposure—were combined for each group (i.e., non-exposed/non-diabetic (V(−)ND), non-exposed/diabetic (V(−)DB), CV-exposed/non-diabetic (V(+)ND), and CV-exposed/diabetic (V(+)DB)) and analyzed.

### 3.2. Effects of CVB4 Exposure and Diabetes on Several Physiologic Parameters

**Figure 2:**
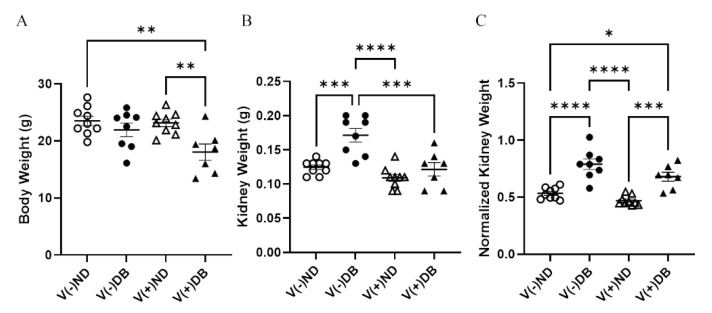
Body weight, kidney weight, and normalized kidney weight: (**A**) body weight, (**B**) kidney weight, and (**C**) normalized kidney weight were measured and compared across all four NOD mouse groups (*n* = 7–9; * *p* < 0.05, ** *p* < 0.01, *** *p* < 0.001, **** *p* < 0.0001; 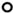 V(−)ND, 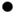 V(−)DB, 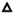 V(+)ND, 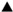 V(+)DB).

Body weight and kidney weight are common measurements taken to assess overall animal and kidney health, respectively. Body weight was significantly lower in V(+)DB animals in comparison to both V(−)ND and V(+)ND animals, while body weight of V(−)DB mice was not significantly different from that of any other group (Figure 2A). In contrast, kidney weight was significantly higher in only V(−)DB mice, in comparison to all other groups (Figure 2B). Kidney weight normalized by body weight was significantly higher in both groups of DB mice relative to both ND control groups, while the two ND groups and the two DB groups did not differ between themselves (Figure 2C). 

To assess kidney function differences between each group, UACR and SrCr were measured. UACR and SrCr were not affected by either CVB4 exposure or development of T1D (Supplementary Figure S1). 

### 3.3. The Effect of Hyperglycemia on CVB4 Levels in the Kidneys

**Figure 3:**
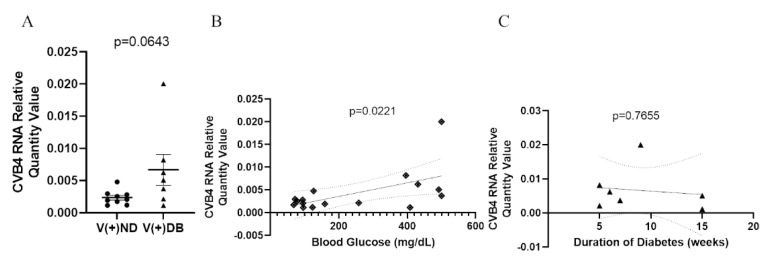
CVB4 abundance in exposed NOD mice analyzed by diabetic status: (**A**) Comparison of CVB4 RNA abundance between exposed/non-diabetic and exposed/diabetic mice. Data were normalized by the geometric mean of GAPDH and γ-actin. CVB4 RNA abundance was plotted against (**B**) blood glucose levels and (**C**) duration of diabetes (*n* = 7–9; 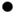 V(−−)DB, 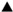 V(+)DB, 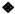 all diabetic mice); 95% confidence intervals are represented by dashed lines.

Previous reports in NOD mice have demonstrated that diabetic animals have a reduced ability to clear *Staphylococcus aureus* in a model of diabetic foot infections when compared to non-diabetic mice [59]. Moreover, the onset of diabetes development in NOD mice is well accepted to be influenced by the onset of insulitis in the pancreas, as well as defects in antigen presentation, T-cell response, cytokine production, and general immune modulation, all of which are involved in pathogen detection and clearance. We have previously demonstrated that CVB4-exposed NOD mice had measurable amounts of CVB4 RNA in the kidneys 17 weeks after initial exposure, although the peak was measured at 3 days [33]. Therefore, we measured CVB4 RNA abundance in CVB4-exposed non-diabetic and diabetic animals to determine whether diabetes (or the diabetic milieu) plays a role in CVB4 levels in the kidney. CVB4 RNA abundance was assessed via RT-qPCR, comparing CVB4-exposed ND and DB NOD mouse kidneys. CVB4 RNA abundance tended to be elevated in animals that had developed T1D (*p* = 0.0643) (Figure 3A). In addition, Pearson’s correlation coefficient revealed a significant positive correlation between CVB4 RNA abundance and blood glucose levels (*p* = 0.0221) (Figure 3B), but not duration of diabetes at the time of euthanasia (*p* = 0.7655) (Figure 3C).

### 3.4. Pattern Recognition Receptor (PRR) Response 

**Figure 4:**
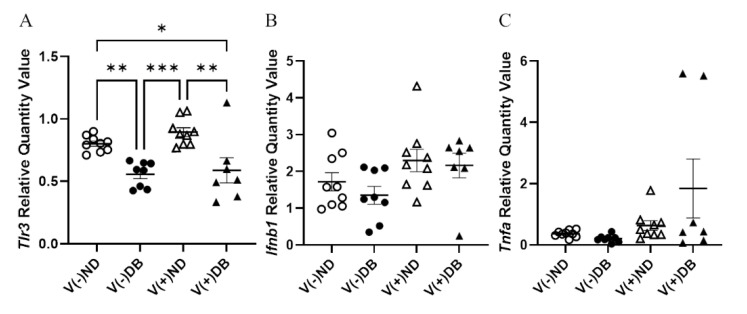
PRR response: (**A**) *Tlr3*, (**B**) *Ifnβ*, and (**C**) *Tnfα* were measured by RT-qPCR and compared across all four NOD mouse groups. Data were normalized by the geometric mean of *Gapdh* and *Actg1* (*n* = 7–9; * *p* < 0.05, ** *p* < 0.01, *** *p* < 0.001; 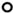
V(−)ND, 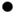
V(−)DB, 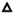
V(+)ND, 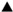
V(+)DB).

While prolonged CVB4 exposure occurred at similar levels in both diabetic and non-diabetic animals, their responses to exposure varied; thus, we assessed pattern recognition receptor (PRR) activity in CVB4-exposed non-diabetic and diabetic groups. Previously, we demonstrated that a number of pattern recognition response receptors and pathway members were significantly upregulated three days after inoculation [33]. As representatives of these genes, gene expression of *Tlr3*—a known PRR pathway member that responds to CVB4 [60,61]—and other PRR signaling products, *Ifnb1* and *Tnfa*, was assessed by RT-qPCR in the current study. *Tlr3* expression was significantly lower in DB kidneys compared to ND kidneys, while CVB4 exposure did not appear to play a role in *Tlr3* expression (compare V(−)ND with V(+)ND and V(−)DB with V(+)DB) (Figure 4A). *Ifnb1* and *Tnfa* expression was not significantly different among any of the groups; however, *Tnfa* expression did vary widely in the V(+)DB group, with an 11-fold and 76-fold difference between the highest and lowest *Ifnb1*- and *Tnfa*-expressing kidneys, respectively (Figure 4C). 

### 3.5. Histopathological Changes after CVB4 Exposure and Diabetes Development

**Figure 5:**
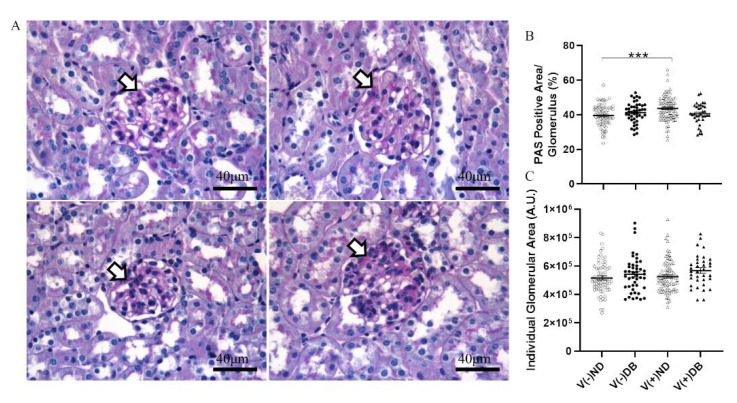
Histological evaluation of kidneys with and without CVB4 exposure and diabetes: (**A**) Representative images of periodic acid–Schiff staining of non-exposed (upper two panels) and CVB4-exposed (lower two panels) NOD mouse kidneys without (left two panels) and with diabetes (right two panels). Plots of (**B**) PAS-positive area and (**C**) glomerular area were plotted per individual glomerulus. PAS-positive area was normalized by dividing dark pink (PAS) staining by glomerular area and multiplying by 100 for each glomerulus. Arrowheads point to dark pink PAS-positive areas. Images taken at 400× magnification (*n* = 30–65; *** *p* < 0.001; 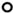
V(−)ND, 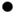
V(−)DB, 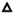
V(+)ND, 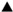
V(+)DB).

We previously established that CVB4 exposure in non-diabetic NOD mice resulted in elevated glomerular periodic acid–Schiff (PAS) staining 17 weeks after CVB4 exposure compared to non-exposed mice [33]. To determine whether the PAS-positive area, indicative of chronic histological alterations such as basement membrane thickening and mesangial expansion, was increased in the kidneys of non-exposed mice with T1D or mice with both previous CVB4 exposure and T1D, PAS staining of non-exposed and CVB4-exposed diabetic NOD mouse kidneys was included in an expanded analysis, along with the previous measurements. PAS-positive areas were observed in glomeruli throughout the kidneys in all four NOD mouse groups (Figure 5A). The glomerular area and percent PAS-positive area of each glomerulus were quantified in five randomly selected glomeruli from each kidney section (Figure 5B,C). The only significant difference observed was in the percent PAS-positive area between V(−)ND and V(+)ND, following CVB4 exposure in the absence of diabetes, as reported previously [33]; a similar increase was not seen for the diabetic groups. The absence of any difference in glomerular area between any of the groups demonstrates that these findings result solely from an increase in the PAS-positive area, and not from a decrease in glomerular size, as also reported previously [33]. 

Immune cell infiltration and collagen deposition have been identified previously in the kidneys of both DB NOD mice and CV-exposed (ND) mice [39,62,63]; therefore, we assessed the presence of immune cell infiltrates and collagen deposition in the kidneys of all four NOD mouse groups. Immune cell infiltration was observed in peri-arteriolar regions of the kidney via PAS staining, regardless of diabetes or viral exposure status (Supplementary Figure S2). There was no difference between any of the NOD mouse groups in terms of total infiltration area or number of infiltration locations observed at the time of euthanasia. Trichrome staining was performed to assess renal fibrosis in the presence or absence of CVB4 exposure and/or T1D. No observable collagen deposition was observed in NOD mouse kidneys in any group (Supplementary Figure S3).

### 3.6. Effect of CVB4 Exposure and Diabetes on Molecular Markers of Fibrosis

**Figure 6:**
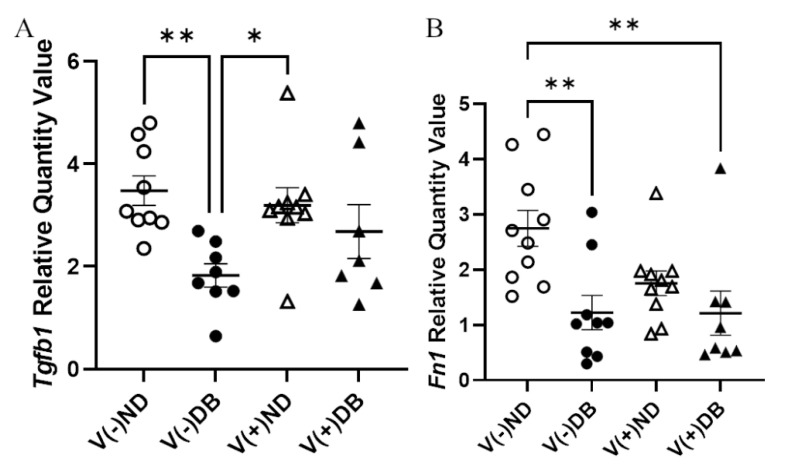
Fibrosis pathway gene expression: (**A**) *Tgfβ1* and (**B**) *Fn1* were measured by RT-qPCR and compared across all four NOD mouse groups. Data were normalized by the geometric mean of *Gapdh* and *γ-actin* (*n* = 7–9; * *p* < 0.05, ** *p* < 0.01; 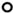
V(−)ND, 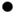
V(−)DB, 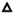
V(+)ND, 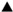
V(+)DB).

Although no trichrome-positive staining was observed, RNA expression of *Tgfb1* and *Fn1* was evaluated by RT-qPCR to assess fibrosis pathway activation with or without CVB4 exposure and/or T1D. *Tgfb1* expression was significantly lower in V(−)DB as compared to V(−)ND and V(+)ND NOD mouse kidneys, while expression in V(+)DB NOD mouse kidneys did not differ significantly from any other group (Figure 6A). *Fn1* expression was significantly reduced in V(−)DB and V(+)DB NOD mouse kidneys as compared to V(−)ND NOD mouse kidneys, while *Fn1* expression in V(+)ND NOD mouse kidneys was not significantly different from any other group (Figure 6B). CVB4 exposure alone had no effect on *Tgfb1* or *Fn1* expression (compare V(−)ND to V(+)ND and V(−)DB to V(+)DB mice, respectively) (Figure 6A,B).

### 3.7. Effects of CVB4 Exposure and Diabetes on Kidney Injury Biomarkers

**Figure 7:**
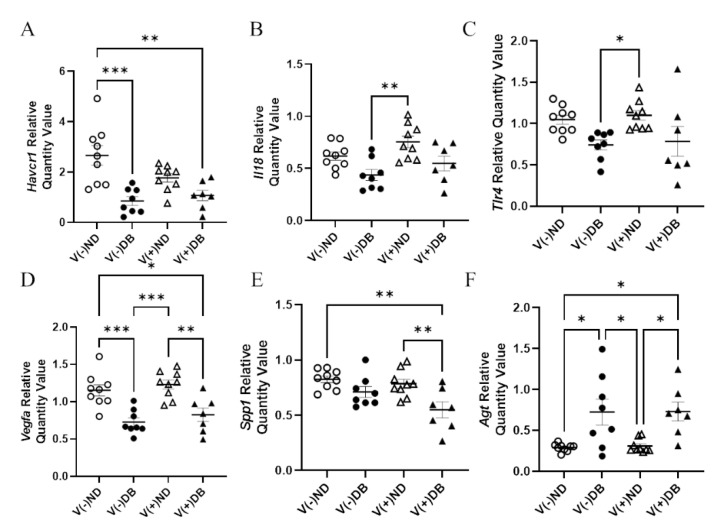
Kidney injury biomarkers: (**A**) *Havcr1*, (**B**) *Spp1*, (**C**) *Agt*, (**D**) *Vegfa*, (**E**) *Il18*, and (**F**) *Tlr4* were measured by RT-qPCR and compared across all four NOD mouse groups. Data were normalized by the geometric mean of *Gapdh* and *γ-actin* (*n* = 7–9; * *p* < 0.05, ** *p* < 0.01, *** *p* < 0.001; 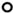
V(−)ND,
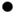
V(−)DB, 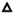
V(+)ND, 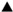
V(+)DB).

In order to determine molecular distinctions between each treatment group, known kidney injury biomarkers [53,64] were also evaluated by RT-qPCR for each NOD mouse group. RNA expression of *Ccl2*, *Cd68*, *Il6*, *Cxcl10*, *Mhc1*, *Lcn2*, *Nos2*, and *Tlr4* showed no significant differences in expression between any of the groups (data not shown). Gene expression of *Havcr1* was significantly lower in V(−)DB and V(+)DB kidneys when compared to V(−)ND, while V(+)ND was at an intermediate level (Figure 7A). *Spp1* was significantly lower in V(+)DB when compared to V(−)ND and V(+)ND, while V(−)DB was at an intermediate level (Figure 7B). *Agt* was significantly higher and *Vegfa* was significantly lower in DB kidneys when compared to the ND groups (Figure 7C,D). *Il18* and *Tlr4* expression was significantly higher in V(+)ND mice compared to V(−)DB mice, whereas both V(−)ND and V(+)DB were not different from any other group (Figure 7E,F). *Agt*, *Vegfa*, *Il18*, and *Tlr4* were the only biomarkers measured that were significantly different between the individual injury (V(−)DB and V(+)ND) mouse groups. 

### 3.8. Correlation of CVB4 Abundance and Blood Glucose with Body Weight, Kidney Weight, and Gene Expression

Although RNA expression of several kidney injury biomarkers and pattern recognition response genes, body weight, kidney weight, normalized kidney weight, and PAS-positive area all demonstrated the potential to differentiate groups by treatment, correlation coefficients were calculated to determine whether additional measured parameters may be influenced by CVB4 exposure or hyperglycemia. Pearson’s correlation coefficients were calculated, and significant correlation coefficients are listed in Table 1. CVB4 abundance was negatively correlated with kidney weight, body weight, and *Spp1* expression, and positively correlated with *Tnfa*, *Ccl2*, *Lcn2*, *Nos2*, and *Mhc1* RNA expression. Blood glucose was negatively correlated with body weight, *Tgfb1*, *Fn1*, *Havcr1*, *Il18*, *Spp1*, *Vegfa*, *Tlr4*, and *Tlr3* RNA expression, and positively correlated with average glomerular size, kidney weight, normalized kidney weight, UACR, and *Agt* RNA expression. While kidney weight, body weight, and *Spp1* RNA expression were correlated with both CVB4 abundance and blood glucose, kidney weight correlation was uniquely negatively correlated with one and positively correlated with the other. *Tnfa*, *Ccl2*, *Lcn2*, *Nos2*, and *Mhc1* expression were correlated only with CVB4 abundance, while average glomerular size, normalized kidney weight, UACR, *Tgfb1*, *Fn1*, *Havcr1*, *Il19*, *Agt*, *Vegfa*, *Tlr4*, and *Tlr3* expression were correlated only with blood glucose.

### 3.9. Identification of Kidney Injury Profiles

**Figure 8:**
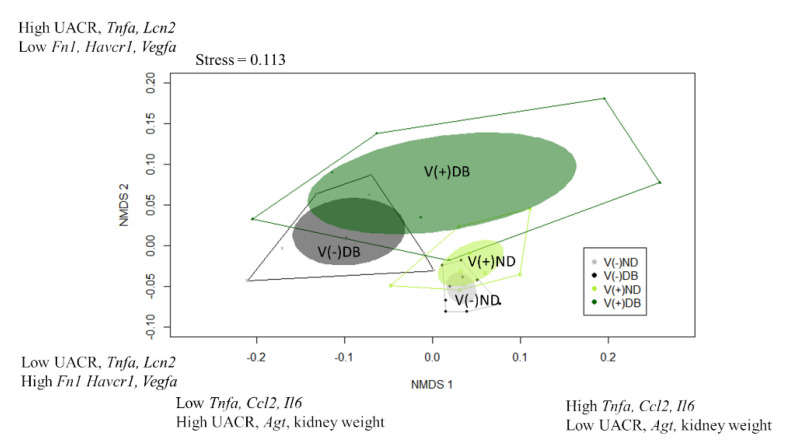
Non-metric multidimensional scaling of kidney injury biomarkers: Gene expression data were combined with physiological data to create a dissimilarity matrix used to visualize the similarities and differences between the four NOD mouse groups. Using two lines, the variation within the data was captured well, with a stress close to 0.1 (0.113). Plotted are NMDS1 vs. NMDS2. Solid dots represent individual animals, solid colored ellipses represent the 95% confidence interval, and open polygons represent 100% of the data. The top three drivers of group separation are noted on each axis.

While the correlation coefficients calculated in Table 1 suggest which parameters are influenced by CVB4 abundance and blood glucose levels, they only consider the main effects of treatment (CVB4 or glucose); thus, when a factor is correlated with both CVB4 abundance and blood glucose (i.e., kidney weight, body weight, and *Spp1* expression), it is unclear which is the driving factor (CVB4 or glucose). Moreover, all analyses performed thus far (Figure 2, Figure 3, Figure 4, Figure 5, Figure 6 and Figure 7) are single-parameter measurements, limiting our ability to determine differences between each treatment group. For this reason, we utilized a non-parametric approach that would allow us to consider all measurements thus far in the same analysis, thereby allowing us to identify which treatment groups are the most similar and dissimilar, and the driving factors for group separation. Non-metric multidimensional scaling (NMDS) was performed to visualize the groups in a multidimensional space (Figure 8). Data included in this analysis included everything used for the Pearson’s correlation coefficients, with the exception of average PAS-positive area, average glomerular size, number of areas infiltrated, and sum of area of immune infiltrates, which were not analyzed at seven weeks. In addition, CVB4 abundance and blood glucose were not included due to the desire to divide the animals into groups based on “profiles” demonstrating effects of CVB4 exposure and diabetes, and not dividing directly by levels of CVB4 RNA and glucose. NMDS1 demonstrated complete group separation between V(−)ND and V(−)DB, and nearly complete group separation between V(+)ND and V(−)DB. However, there was no group separation between V(−)ND and V(+)ND, between V(−)DB and V(+)DB, or between V(+)ND and V(+)DB. V(−)ND took up the least amount of space on NMDS1, with V(+)ND and V(−)DB taking up a moderate amount of space, and V(+)DB taking up the most space, completely overlapping all groups (Figure 8). The top three positive drivers of NMDS1 variability were *Tnfa*, *Ccl2*, and *Il6*, while the top three negative drivers were UACR, *Agt*, and kidney weight. Interestingly, four of these were not significantly different in any of the NOD mouse groups when analyzed individually. NMDS2 demonstrated a downward shift in ND animal groups compared to an upward shift in DB animal groups. Group shifting was also observed between V(−)ND (shifted down) and V(+)ND (shifted up), and between V(−)DB (shifted down) and V(+)DB (shifted up), with the two diabetic groups taking up a larger space on NMDS2 compared to the two ND groups. Moreover, the group shifted the lowest on NMDS2 was V(−)ND, with V(−)DB and V(+)ND being mid-range groups, and the highest shift being V(+)DB. The top three positive drivers for group separation for NMDS2 were UACR, *Tnfa*, and *Lcn2*, while the top three negative drivers were *Fn1*, *Havcr1*, and *Vegfa*. Similar to NMDS1, three drivers of NMDS2 were not significantly different in any of the NOD mouse groups when measured individually. The complete list of NMDS1 and NMDS2 rankings can be found in Supplementary Table S4. A PERMANOVA followed by a pairwise PERMANOVA post-hoc test revealed that there were significant differences between (1) V(−)ND and both diabetic groups (V(−)DB and V(+)DB), and (2) the V(−)DB and V(+)DB groups. There were no significant differences between V(+)ND and V(−)DB, or between V(+)ND and any other group.

## 4. Discussion

Current measures for assessing kidney function only provide insight into how severe the kidney damage is and, thus, poorly predict the cause(s) of kidney damage. With the recognition that multiple insults may be involved concomitantly in kidney injury, we sought to determine insult-specific kidney injury profiles unique to diabetes (the number one cause of ESRD) and CVB4 infection (a viral infection that can also lead to T1D and kidney damage), in order to improve our understanding of which patients are more at risk of developing kidney injury and, eventually, progressing to ESRD. Thus, this study briefly outlines the contributions of a one-time CVB4 exposure and T1D, both alone (single-insult contributions) and together (double-insult contributions), on the development of kidney injury and the phenotypic similarities and differences between each treatment.

This study provides supporting evidence that measures of kidney injury likely vary based on the type of injury. For example, elevated normalized kidney weight and *Agt* RNA expression, along with decreased *Tlr3* and *Vegfa* RNA expression, demonstrated that diabetes, but not CVB4 exposure, was responsible for their differential expression. In comparison, none of the measurements in this study demonstrated that CVB4 exposure alone was responsible for their differential expression—in other words, with significant difference occurring in V(+) animals as compared to V(−) animals, regardless of the diabetes status. Additionally, PAS-positive area was only different in the single-insult CVB4 (V(+)ND) animals, while kidney weight and *Tgfb1* expression differed in the single-insult T1D (V(−)DB) animals, and body weight in the double-insult (V(+)DB) group. Furthermore, these data demonstrate that some measures of kidney health can differ between CVB4 exposure and T1D, either individually or together.

Additional evidence for differing kidney phenotypes in each group comes from the levels of CVB4 identified in the kidneys. While there was no significant difference in CVB4 abundance between the CVB4-exposed ND and DB groups, there was a significant correlation between CVB4 abundance and blood glucose. This correlation supports the findings of a previous study demonstrating that diabetic NOD mice have an impaired ability to clear *Staphylococcus aureus* infection when compared to non-diabetic counterparts [59]. Interestingly, the PRR response—as measured by *Tlr3*, *Ifnb1*, and *Tfna* RNA expression—was downregulated or unchanged with DB regardless of CVB4 exposure. Together, these data suggest that CVB4 exposure may set up a chronic inflammatory milieu that, in some instances, is dependent on T1D (i.e., *Tlr3*).

While, together, the aforementioned measurements demonstrate that each of the four groups could be differentiated by phenotype, it is difficult to determine by any one measurement which group a single mouse may fall into. For this reason, we combined all measurements, with the addition of well-defined kidney injury biomarkers, to determine whether interactions may better help identify profiles unique to each group. A permutational multivariate analysis of variance (PERMANOVA) supported the finding that there was significant group separation between treatments, with the post-hoc PERMANOVA identifying significant differences between (1) V(−)ND and V(−)DB, (2) V(−)ND and V(+)DB, and (3) V(−)DB and V(+)DB. We can only speculate that there was no identified group separation between non-CVB4-exposed and CVB4-exposed single-insult groups (V(−)DB and V(+)ND), because we did not identify and measure the major factors that would contribute to separation of these groups. While the groups were not all significantly different, these data suggest that additional analyses using a broader scale approach—such as RNA sequencing—may provide higher resolution to each group, and a better understanding of which genes are responsible for defining each group. 

In addition to determining group separation, NMDS allowed us to identify the drivers that were largely responsible for group separation. The top three positive and top three negative drivers explaining the variance captured on NMDS1 (Figure 8) were *Tnfa*, *Ccl2*, *Il6*, UACR, *Agt*, and kidney weight, respectively, while the top three positive and top three negative drivers separating NMDS2 were UACR, *Tnfa*, *Lcn2*, *Fn1*, *Havcr1*, and *Vegfa*, respectively. Interestingly, some of the drivers for group separation (i.e., *Tnfa*, *Ccl2*, *Il6*, *Lcn2*, and UACR) were not significantly different between any of the four mouse groups when analyzed in isolation, but are widely known indicators of kidney injury [23,53,64]. This suggests that within each mouse group, there may be a larger degree of variability in kidney phenotypes in response to insult than previously thought, and while a gene’s expression may not be directly responsible for group separation, including genes that have no significant differences between groups in the analysis may provide insight into potential interaction effects. Shedding light on additional responses to injury will allow us to further understand how the kidney responds to an insult and results in subsequent injury or repair. 

Taken together, this study provides evidence that different kidney insults can result in different kidney injury profiles and subsequent health outcomes. Identifying unique kidney injury biomarkers or combinations of biomarkers for each group could be used to suggest the origin and mechanism of the kidney injury, as well as novel treatment approaches, with little prior knowledge of patient history. While proving the role that each biomarker plays in kidney injury may not be attainable, at the very least, unique kidney injury profiles will help us take strides toward more personalized medicine approaches for treating kidney disease. 

## Figures and Tables

**Table 1:** Significant Pearson’s correlation coefficients between various measured parameters and CVB4 abundance and blood glucose levels (*p* < 0.05).

Parameter Measured	CVB4 Abundance	Blood Glucose
Average PAS-Positive Area		
Average Glomerular Size		0.4739
Number of Areas Infiltrated		
Sum of Area of Immune Infiltrates		
Duration of Diabetes		
Body Weight	−0.5702	−0.5103
Kidney Weight	−0.3914	0.5443
Normalized Kidney Weight		0.8446
UACR		0.4242
SrCr		
*Tgfb1* Expression		−0.4909
*Fn1* Expression		−0.4681
*Tnfa* Expression	0.7409	
*Ifnb* Expression		
*Il6* Expression		
*Havcr1* Expression		−0.6175
*Ccl2* Expression	0.4346	
*Cd68* Expression		
*Lcn2* Expression	0.7907	
*Il18* Expression		−0.5747
*Spp1* Expression	−0.5143	−0.5142
*Agt* Expression		0.7034
*Vegfa* Expression		−0.7406
*Cxcl10* Expression		
*Nos2* Expression	0.5168	
*Mhc1* Expression	0.7696	
*Tlr4* Expression		−0.5542
*Tlr3* Expression		−0.6874

## Data Availability

The data presented in this study are openly available in Dryad at https://doi.org/10.5061/dryad.547d7wm8r.

## Supplementary Materials

The following are available online at https://www.mdpi.com/article/10.3390/microorganisms9112357/s1: Figure S1: Urine albumin and creatinine excretion and serum creatinine; Figure S2: Immune cell infiltration; Figure S3: Fibrosis evaluation following CVB4 exposure; Table S1: SYBR green primer names, sequences, and references; Table S2: TaqMan primer names and catalog numbers; Table S3: Sample size and duration of diabetes per NOD mouse group at euthanasia; Table S4: NMDS1 and NMDS2 species scores for each parameter.
