# Supplementary material for: Consequences of Both Coxsackievirus B4 and Type 1 Diabetes on Female Non-Obese Diabetic Mouse Kidneys
Source: Microorganisms. 2021 Nov 15;9(11):2357. doi: 10.3390/microorganisms9112357 (PMC8623636; doi:10.3390/microorganisms9112357)
Supplement: Supplementary file 1 [file microorganisms-09-02357-s001.zip › microorganisms-1383506-supplementary.pdf]

Article

Consequences of Both Coxsackievirus B4 and Type 1 Diabetes on Female Non-Obese Diabetic Mouse Kidneys

Debra L. Walter [1,2,*], Jean R. Thuma [3], Ramiro Malgor [1,4,5], Frank L. Schwartz [3,5], Kelly D. McCall [1,2,3,4,5,6,7,†] and Karen T. Coschigano [1,4,5,†]

1 Interdisciplinary Program in Molecular and Cellular Biology, Ohio University, Athens OH, 45701, USA; malgor@ohio.edu (R.M.); mccallk@ohio.edu (K.D.M); coschigk@ohio.edu (K.T.C.)
2 Department of Biological Sciences, College of Arts and Sciences, Ohio University, Athens OH, 45701, USA
3 Department of Specialty Medicine, Heritage College of Osteopathic Medicine, Ohio University, Athens OH, 45701, USA; watts@ohio.edu (J.R.T.); endoguy.ou@gmail.com (F.L.S.)
4 Department of Biomedical Sciences; Heritage College of Osteopathic Medicine, Ohio University, Athens OH, 45701, USA
5 The Diabetes Institute, Heritage College of Osteopathic Medicine, Ohio University, Athens OH, 45701, USA
6 Translational Biomedical Sciences Program, Heritage College of Osteopathic Medicine, Ohio University, Athens OH, 45701, USA
7 Biomedical Engineering Program, Russ College of Engineering and Technology, Ohio University, Athens OH, 45701, USA
* Correspondence: debwalter@vt.edu
† Denotes shared senior authorship

Supplementary Materials

Supplementary Table S1. SYBR green primer names, sequences and references.

Gene Name	Full Name	Strand	Sequence	Reference
**CVB4**	Coxsackievirus B4	+<br>−	CCCACAGGACGCTCTAATA<br>CAGAGTTACCCGTTACGACA	[1]
Tlr3	Toll Like Receptor 3	+<br>−	AATCCTTGCGTTGCGAAGTG<br>GGTTCAGTTGGGCGTTGTTC	[2]
Ifnb1	Interferon Beta	+<br>−	ATAAGCAGCTCCAGCTCCAA<br>CTGTCTGCTGGTGGAGTTCA	[2]
Tnfa	Tumor Necrosis Factor Alpha	+<br>−	CGGTCCCCAAAGGGATGAG<br>CCTTGAAGAGAACCTGGGAGTA	[2]
Tgfb1	Transforming Growth Factor Beta	+<br>−	TTGCTTCAGCTCCACAGAGA<br>CGGGTTGTGTTGGTTGTAGA	[2]
Fn1	Fibronectin	+<br>−	CTTTGGCAGTGGTCATTTCA<br>TGGTAGGTCTTCCCATCGTC	[2]
Havcr1	Hepatitis A Virus Cellular Receptor 1	+<br>−	TCCACACATGTACCAACATCAA<br>TGTCTCATGGGGACAAAATG	[2]
Il6	Interleukin 6	+<br>−	GATGGATGCTACCAAACTGGA<br>GGAAATTGGGGTAGGAAGGA	[2]
Gapdh	Glyceraldehyde 3-phosphate dehydrogenase	+<br>−	TGTGTCCGTCGTGGATCTGA<br>CCTGCTTCACCACCTTCTTGA	[3]
Actg1	Actin Gamma 1	+<br>−	ACCAACAGCAGACTTCCAGGAT<br>AGACTGGCAAGAAGGAGTGG-TAA	[4]
Cd68	CD68 Molecule	+<br>−	CTTCCCACAGGCAGCACAG<br>AATGATGAGAGGCAGCAA-GAGG	[3]

Supplementary Table S2. TaqMan primer names and catalog numbers.

Gene Name	Full Name	Supplier	Catalog Number
Spp1	Osteopontin	Thermo Fisher, Waltham, MA, USA	Mm00436767_m1
Agt	Angiotensinogen	Thermo Fisher, Waltham, MA, USA	Mm00599662_m1
Vegfa	Vascular Endothelial Growth Factor A	Thermo Fisher, Waltham, MA, USA	Mm01281449_m1
Il18	Interleukin 18	Thermo Fisher, Waltham, MA, USA	Mm00434226_m1
Tlr4	Toll Like Receptor 4	Thermo Fisher, Waltham, MA, USA	Mm00445273_m1
Ccl2	X-C Motif Chemokine Ligand 2	Thermo Fisher, Waltham, MA, USA	Mm00441242_m1
Lcn2	Lipocalin 2	Thermo Fisher, Waltham, MA, USA	Mm01324470_m1
Cxcl10	C-X-C Motif Chemokine Ligand 10	Thermo Fisher, Waltham, MA, USA	Mm00445235_m1
Nos2	Nitric Oxide Synthase 2	Thermo Fisher, Waltham, MA, USA	Mm00468487_m1
Mhc1	Major Histocompatibility Complex 1	Thermo Fisher, Waltham, MA, USA	Mm01309902_m1

Supplementary Table S3. Sample size and duration of diabetes per NOD mouse group at euthanasia.

Group†	7 Weeks Post Exposure	Weeks Diabetic‡	12 Weeks Post Exposure	Weeks Diabetic‡	17 Weeks Post Exposure	Weeks Diabetic‡
V(−) ND	3		3		3	
V(−) DB	3	2,3,7	3	1,6,7	2	3,3
V(+) ND	3		3		3	
V(+) DB	1	5	4	5,7,8,9	2	14,14

† V(−) ND = Non-Exposed/Non-Diabetic; V(−) DB = Non-Exposed/Diabetic; V(+) ND = Virus-Exposed/Non-Diabetic; V(+) DB = Virus-Exposed/Diabetic.‡ Measured by the number of glucose readings above 250mg/dL prior to euthanasia

Supplementary Table S4. NMDS1 and NMDS2 species scores for each parameter.

Gene Name	MDS1	MDS2
Fn1	0.078974	−0.10507
Havcr1	0.094793	−0.09177
Vegfa	0.008206	−0.04695
Body Weight	−0.07569	−0.04397
Spp1	−0.04766	−0.04079
Tlr4	0.025125	−0.03933
Tlr3	0.003621	−0.03766
Tgfb	0.063486	−0.03173
Nos2	0.057767	−0.02846
Il18	0.004907	−0.02832
Kidney Weight	−0.12466	−0.00782
SrCr	−0.09726	0.010299
Cd68	0.073767	0.01456
Ccl2	0.197826	0.027797
Normalized Kidney Weight	−0.11768	0.030018
Ifnb	0.054015	0.031058

Mhc1	0.016621	0.043313
Il6	0.181237	0.043341
Cxcl10	−0.00561	0.096404
Agt	−0.15425	0.11908
Lcn2	0.125138	0.124897
Tnfa	0.291052	0.143158
UACR	−0.1869	0.158651

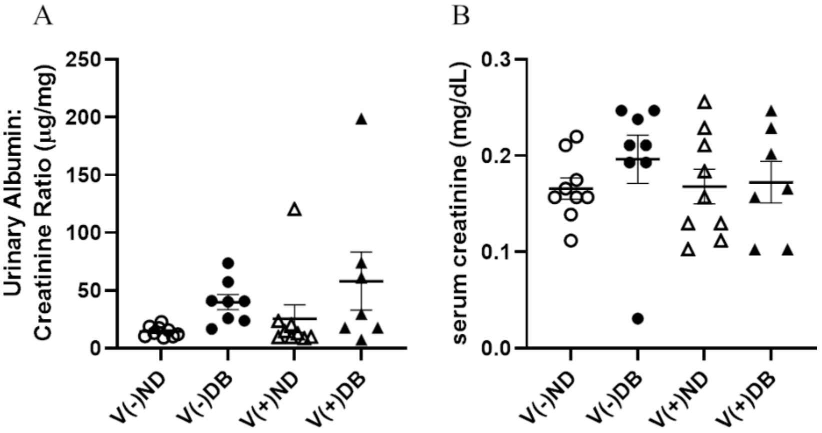

Supplementary Figure S1. Urine albumin and creatinine excretion and serum creatinine levels. (**A)** UACR and (**B**) serum creatinine were measured and compared across all four NOD mouse groups (*n* = 7–9; ○V(−)ND,●V(−)DB,△V(+)ND, ▲V(+)DB).

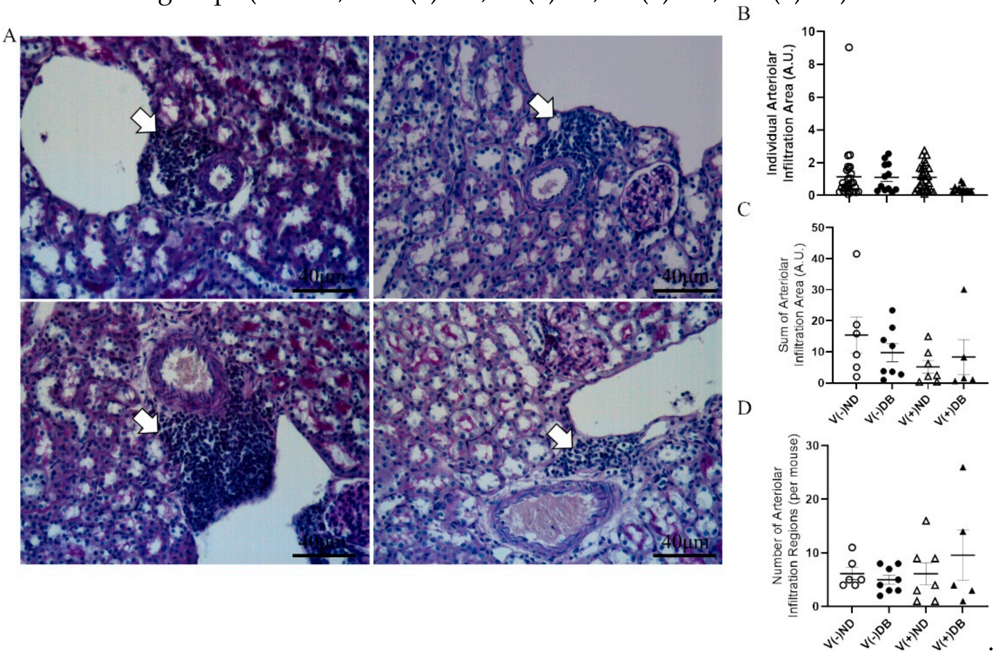

Supplementary Figure S2. Immune Cell Infiltration. (**A**) Representative images of Periodic acid-Schiff staining of non-exposed (upper two panels) and CVB4-exposed (lower two panels) NOD mouse kidneys without (left two panels) and with diabetes (right two panels). Plots of infiltration (**B**) individual areas, (**C**) area sum and (**D**) number of locations per three kidney sections per mouse. Arrowheads point to immune cell infiltration. Images taken at 200× magnification (*n* = 30–65; ○ V(−)ND,●V(−)DB,△V(+)ND, ▲V(+)DB).

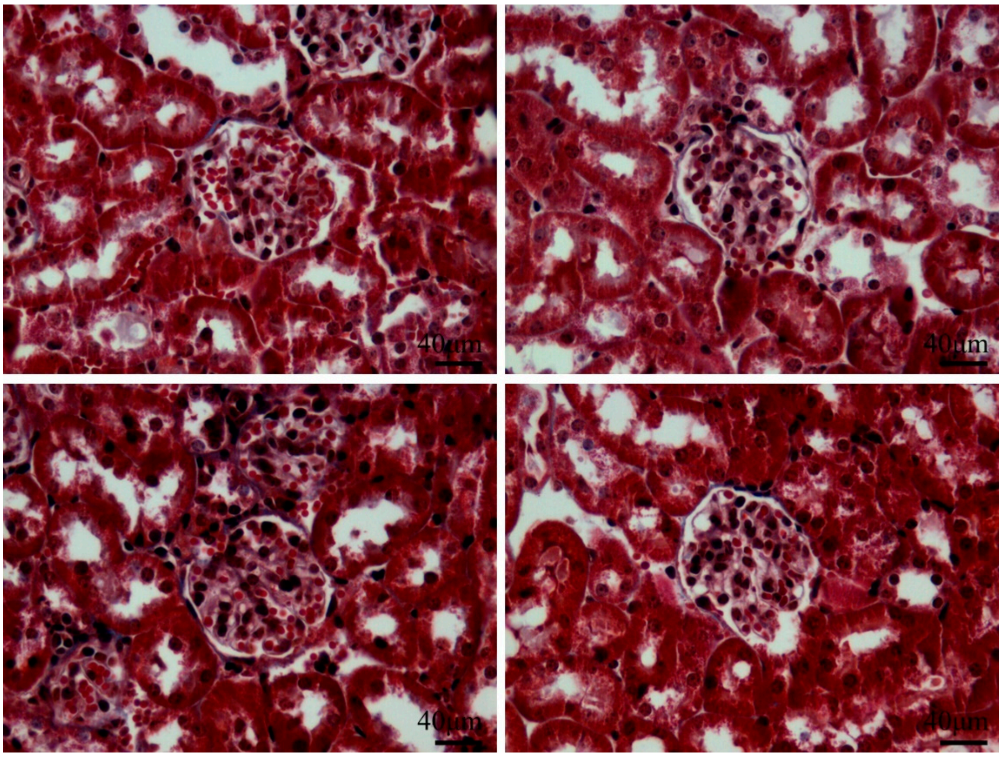

Supplementary Figure S3. Fibrosis evaluation following CVB4 exposure. Representative images of Masson's Trichrome stain of non-exposed (upper two panels) and CVB4-exposed (lower two panels) NOD mouse kidneys without (left two panels) and with diabetes (right two panels). Images taken at 400× magnification.

Reference

1. Walter, D.L.; Benner, S.E.; Oaks, R.J.; Thuma, J.R.; Malgor, R.; Schwartz, F.L.; Coschigano, K.T.; McCall, K. Coxsackievirus B4 Exposure Results in Variable Pattern Recognition Response in the Kidneys of Female Non-Obese Diabetic Mice Before Establishment of Diabetes. *Viral Immunol.* **2020**, *33*, 494–506, doi:10.1089/vim.2019.0188.
2. Ye, J.; Coulouris, G.; Zaretskaya, I.; Cutcutache, I.; Rozen, S.; Madden, T.L. Primer-BLAST: A Tool to Design Target-Specific Primers for Polymerase Chain Reaction. BMC Bioinformatics **2012**, *13*, 134, doi:10.1186/1471-2105-13-134.
3. Fujimoto, M.; Maezawa, Y.; Yokote, K.; Joh, K.; Kobayashi, K.; Kawamura, H.; Nishimura, M.; Roberts, A.B.; Saito, Y.; Mori, S. Mice Lacking Smad3 Are Protected against Streptozotocin-Induced Diabetic Glomerulopathy. Biochem. Biophys. Res. Commun. **2003**, *305*, 1002–1007, doi:10.1016/s0006-291x(03)00885-4.
4. Lefever, S.; Vandesompele, J.; Speleman, F.; Pattyn, F. RTPrimerDB: The Portal for Real-Time PCR Primers and Probes. Nucleic Acids Res. **2009**, *37*, D942-945, doi:10.1093/nar/gkn777.
